# Metabolic fate of fructose in human adipocytes: a targeted ^13^C tracer fate association study

**DOI:** 10.1007/s11306-014-0716-0

**Published:** 2014-08-03

**Authors:** Vijayalakshmi Varma, László G. Boros, Greg T. Nolen, Ching-Wei Chang, Martin Wabitsch, Richard D. Beger, Jim Kaput

**Affiliations:** 1 Division of Systems Biology, National Center for Toxicological Research, FDA, 3900 NCTR Road, Jefferson, AR 72079 USA; 2 SiDMAP LLC, Los Angeles, CA 90064 USA; 3 Los Angeles Biomedical Research Institute (LABIOMED), Harbor-UCLA Medical Center, Torrance, CA 90502 USA; 4 Department of Pediatrics, Harbor-UCLA Medical Center, Torrance, CA 90502 USA; 5 Division of Bioinformatics and Biostatistics, National Center for Toxicological Research, Jefferson, AR 72079 USA; 6 Division of Pediatric Endocrinology and Diabetology, University of Ulm, Ulm, Germany; 7 Systems Nutrition and Health, Nestle Institute of Health Sciences, Lausanne, Switzerland

**Keywords:** Human adipocytes, Fructose metabolism, ^13^C labeled fructose, Isobolomics, Targeted tracer fate association study, Fatty acids

## Abstract

The development of obesity is becoming an international problem and the role of fructose is unclear. Studies using liver tissue and hepatocytes have contributed to the understanding of fructose metabolism. Excess fructose consumption also affects extra hepatic tissues including adipose tissue. The effects of fructose on human adipocytes are not yet fully characterized, although in vivo studies have noted increased adiposity and weight gain in response to fructose sweetened-beverages. In order to understand and predict the metabolic responses of adipocytes to fructose, this study examined differentiating and differentiated human adipocytes in culture, exposed to a range of fructose concentrations equivalent to that reported in blood after consuming fructose. A stable isotope based dynamic profiling method using [U-^13^C_6_]-d-fructose tracer was used to examine the metabolism and fate of fructose. A targeted stable isotope tracer fate association method was used to analyze metabolic fluxes and flux surrogates with exposure to escalating fructose concentration. This study demonstrated that fructose stimulates anabolic processes in adipocytes robustly, including glutamate and de novo fatty acid synthesis. Furthermore, fructose also augments the release of free palmitate from fully differentiated adipocytes. These results imply that in the presence of fructose, the metabolic response of adipocytes in culture is altered in a dose dependent manner, particularly favoring increased glutamate and fatty acid synthesis and release, warranting further in vivo studies.

**Electronic supplementary material:**

The online version of this article (doi:10.1007/s11306-014-0716-0) contains supplementary material, which is available to authorized users.

## Introduction

Increased and frequent consumption of diets rich in sucrose (table sugar) and/or high fructose corn syrup (sweetener used in confectionaries and many beverages) expose the body to high levels of glucose and fructose. The consumption of sugar and sweeteners has increased for the past 40 years and is thought to be associated with the development of obesity, type II diabetes and cardiovascular diseases (Bray 2004; Brown 2008; Goran et al. 2013; Johnson et al. 2007; Kmietowicz 2012; Sheludiakova et al. 2012). Much attention has been focused on fructose based on its reported effects of increasing circulating triglycerides, dyslipidemia, hyperuricemia, and obesity (Lustig 2010; Tappy and Le 2010). Unlike glucose, fructose is not universally metabolized by all tissues of the body and is not a necessary nutrient for the body. Fructose is primarily and efficiently metabolized by the liver as demonstrated by in vivo and in vitro studies (Elliott et al. 2002; Lim et al. 2010; Mayes 1993; Neuschwander-Tetri 2013). Only insignificant amounts of fructose enter the systemic circulation on consumption of a normal healthy diet. However, the frequent and high consumption of sugar-based beverages and food is reported to causes dyslipidemia, non-alcoholic fatty liver diseases, and other metabolic disorders (Kavanagh et al. 2013; Samuel 2011). High circulating levels of fructose overwhelms the ability of the liver to metabolize the ingested fructose completely, resulting in a spillover of fructose into the systemic circulation (Hui et al. 2009; Munstedt et al. 2011). Excess fructose in the blood is subsequently cleared through the kidneys or it is taken up and metabolized by extra-hepatic tissues, notably adipose, where it may contribute to the development of obesity.

Adipose tissue is a highly active metabolic and endocrine-producing organ (Kershaw and Flier 2004). The metabolic state of the adipose tissue is altered in a dynamic manner in response to the nutritional state by varying and modifying the substances that are taken up or released from it (Frayn et al. 2003; Tan and Vidal-Puig 2008). White adipose tissue can act as a sink of excess energy due to its ability to regulate its own metabolism, either by controlling the differentiation of adipocytes and/or by inducing tissue expansion under conditions of positive energy balance occurring in nutritionally rich states (Frayn et al. 2003). Accumulation and storage of triglycerides can promote the development of obesity. Excess adipose tissue can contribute to increased release of free fatty acids (FFA) into circulation. FFA can deposit ectopically in organs such as skeletal muscle, liver, pancreas and cardiovascular tissue resulting in lipotoxicity and insulin resistance (Axelsen et al. 2010; Snel et al. 2012; Tan and Vidal-Puig 2008).

Fructose metabolism in extra-hepatic tissues is not well studied. Increased fructose levels in the circulation may alter the metabolic characteristics and the function of tissues that metabolize fructose, particularly adipose. Some studies have demonstrated increases in visceral adipose tissue (VAT) volumes and weight gain on exposure to fructose-containing beverages both in human and rodent models (Elliott et al. 2002; Lim et al. 2010; Mayes 1993; Neuschwander-Tetri 2013; Pollock et al. 2012; Ronn et al. 2012). A few earlier studies have examined the metabolism of fructose in rat adipose tissue and some of these studies focused on adipose tissue as a site of extra hepatic fructose metabolism in the presence of hereditary fructose intolerance (Bellido and Herrera 1978; Froesch and Ginsberg 1962). There are no studies to our knowledge that have examined the metabolism and fate of fructose in human adipocytes. Hence, the goal of this study was to characterize the metabolic responses of adipocytes to fructose and to better understand the fate of fructose in adipocytes.

Human Simpson-Golabi-Behmel Syndrome (SGBS) adipocytes (Wabitsch et al. 2001) were used in this study because of its similarities to isolated human adipocytes. To perform this study, a stable isotope based dynamic profiling (SIDMAP) method (Singh et al. 2013) with uniformly labeled fructose [U-^13^C_6_]-d-fructose was used to examine the metabolism and fate of fructose in these adipocytes. The process of adipocyte differentiation is known to entail changes in the metabolite concentrations and alteration of specific pathways including tricarboxylic acid (TCA) cycle, glycolysis, and fatty acid synthesis, between differentiating and differentiated adipocytes (Roberts et al. 2009). Hence, this study examined the responses of adipocytes to fructose at two different time points during adipocyte differentiation including day 8 when adipocytes are predominantly in the differentiating state and at day 16 when the adipocytes were fully differentiated. Based on most common dietary fructose exposures, a broad range of fructose concentrations was examined to predict the metabolic responses of adipocytes to fructose. Additionally, a targeted stable isotope tracer fate association method (Singh et al. 2013) was used to analyze metabolic fluxes using flux surrogates.

## Materials and methods

### Chemicals and reagents

Dexamethasone, 3-isobutyl-1-methylxanthine (IBMX), transferrin, cortisol, triiodothyronine (T3), biotin, pantothenate, dichloromethane, trifluoroacetic anhydride, ethanol, potassium hydroxide (KOH), sodium bicarbonate (NaHCO_3_), HCL, acetone, Dulbecco’s Modified Eagle Medium (DMEM) powder media, Hams’s F12 powder media, fructose and glucose were all obtained from SIGMA Chemical Company (St. Louis, MO, USA). Fetal bovine serum was obtained from Thermo Fisher Scientific (Waltham, MA, USA). Rosiglitazone was obtained from Cayman Chemicals (Ann Arbor, MI, USA), Insulin was obtained from Novo Nordisk [U-^13^C_6_]-d-fructose (>99 % purity, and 99 % isotope enrichment for all carbons) was obtained from Cambridge Isotope Laboratories (Andover, MA, USA), methane and helium (>99.99 % purity), was obtained from PaxAir (Los Angeles, CA, USA), and *n*-butanol was obtained from Regis Chemical. Company (Chicago, IL, USA).

### SGBS cell culture

Human SGBS preadipocytes, that have been described previously (Wabitsch et al. 2001) were kindly provided by Martin Wabitch and used in this study. In brief, the SGBS preadipocytes cells were maintained at 37 °C in a humidified incubator containing a 5 % O_2_ atmosphere. The growth medium consisted of DMEM:F12 (1:1), 33 mM biotin, and 17 mM pantothenate containing 10 % fetal bovine serum and 1 % penicillin–streptomycin. One day post-confluence, the cells were initiated to differentiate into adipocytes by addition of a serum-free differentiation medium containing DMEM with 25 nM dexamethasone, 500 μM IBMX, 2 μM rosiglitazone, 0.01 mg/ml human transferrin, 2 × 10^−8^ M insulin, 10^−7^ M cortisol, 0.2 nM T3, 33 mM biotin, and 17 mM pantothenate. The cells were maintained in differentiation medium for 4 days after which the medium was changed to a serum-free adipogenic medium consisting of DMEM:F12 (1:1) with 0.01 mg/ml human transferrin, 2 × 10^−8^ M insulin, 10^−7^ M cortisol, 0.2 nM T3, 33 mM biotin, and 17 mM pantothenate). Adipogenic medium is essentially similar to the differentiation medium but lacks IBMX, dexamethasone and rosiglitazone. Medium was changed every 2 days from the initiation of differentiation. Cells were allowed to differentiate and harvested after 8 or 16 days. SGBS cells completely differentiate into adipocytes by day 14 of differentiation.

### Fructose treatment of SGBS cells and labeling using U ^13^C6-d-fructose tracer

SGBS preadipocytes were plated at 2 × 10^5^ cells in 60 mm dishes supplemented with 3 ml growth medium (described above) and grown to confluence. All media used for the growth, differentiation and maintenance of adipocytes contained a basal amount of 5 mM glucose, equivalent to the normal blood glucose concentration. In order to determine the effects of fructose exposure to adipocytes at concentrations reported in the systemic circulation following exposure to fructose-rich food (Hui et al. 2009), 0.1, 0.5, 1.0, 2.5, 5, 7.5, 10 mM fructose was added to the media at the initiation of differentiation and maintained in the medium until the collection of cells and medium at end points of either day 8 or until day 16 of differentiation. In order to trace the metabolism of fructose in adipocytes, and to understand the major flow and fate of fructose derived carbons in human adipocytes, a labeled fructose tracer ([U-^13^C_6_]-d-fructose) was utilized. In this experimental paradigm, the labeled fructose was used at 10 % of the respective fructose concentrations. Under such conditions the ^13^C fructose becomes the single tracer of intermediary metabolism by its positional ^13^C carbon contributions to metabolic products. The tracer was added to cells on day 6 and day 14 respectively for a period of 48 h to enable sampling at two different time points, day 8 and day 16 after 48 h. Cell lysates and media were collected for metabolomic assays at endpoints of day 8 and 16. Each concentration was tested in triplicate cultures. For each replicate, samples from two confluent 60 mM dishes were pooled during collection to ensure sufficient material for all assays. At the time of collection, 6 ml of media was first collected centrifuged at 751×*g* for 10 min to remove any dead or floating cells and flash frozen. The cells were rinsed twice with cold PBS and aspirated to remove any remaining medium. 300 μl portion of cold PBS was added to each 60 mm dish, and the cells were scraped and collected, flash frozen and stored at −80 °C until used. Tracer labeled endpoints such as extracellular CO_2_, intra- and extra-cellular glutamine, intra- and extra-cellular fatty acids including palmitate and oleate, lactate, glycogen and ribose, some of which are positionally labeled, were examined as described in the “Materials and methods” and the “Results” sections presented below.

### Targeted ^13^C metabolite studies and metabolic profiling using U ^13^C_6_-d-fructose tracer

**Fig. 1:**
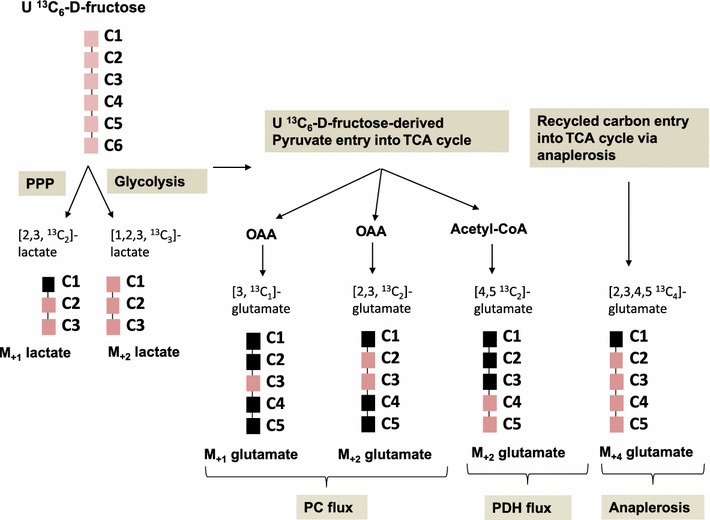
Representation of potential ^13^C labeled lactate and glutamate isotopomers derived from U^13^C_6_-d-fructose used as the single tracer. The *red squares* represent the ^13^C labeled carbons in the isotopomers formed. [2,3 ^13^C_2_] lactate is derived via the pentose phosphate pathway (PPP) while the [1,2,3 ^13^C_3_] lactate is formed via breakdown of U^13^C_6_-d-fructose via glycolysis. [3,^13^C_1_] glutamate and the [2, 3 ^13^C_2_] glutamate are formed from oxaloacetate which is derived from pyruvate via the PC flux. [4,5 ^13^C_2_] glutamate is formed from acetyl-CoA which is derived from pyruvate via the PDH flux. [2,3,4,5 ^13^C4] glutamate is formed from recycled ^13^C labeled carbon entry into TCA cycle via anaplerosis (Color figure online)

Specific extractions were performed as described below and mass spectral data were obtained using a HP5975 N (Agilent, Palo Alto, CA, USA) mass selective detector connected to an HP6890N (Agilent, Palo Alto, CA, USA) gas chromatograph. An Agilent J&W HP-5MS (30 m × 0.25 mm × 0.25 µm; part #: 19091S-433) analytical column was used during glucose, ribose, glutamate, and lactate analyses. For analysis of fatty acids and CO_2_, an Agilent J&W Scientific DB-23 (60 m × 0.25 mm × 0.15 µm; part #: 122-2361) column was used. Helium (>99.99 % purity) was used as the carrier gas in the electron impact ionization (EI) mode and methane (>99.99 % purity, PaxAir, Los Angeles, CA, USA) in the chemical ionization mode. Sample injections were performed using 100 split ratios directly into the heated (250 °C) and pressurized inlet interfaces. Results of mass isotopomers in glutamate are reported as molar fractions where m1, m2, etc. indicate the number of ^13^C atoms in the molecule (Lee et al. 1991). The enrichment, Σmn, is the weighted sum of the labeled species (Σmn = m1 X 1 + m2 X 2 + m3 X 3…) (Marin et al. 2004; Vizan et al. 2005). Lactate was extracted from cell culture media (0.2 ml) by ethylene chloride after acidification with HCl, derivatized to its propylamine–heptafluorobutyrate ester form and applied to the column (Lee et al. 1998). Enrichment of ^13^C-labeled acetyl units derived from [U-^13^C_6_]-fructose in palmitate and oleate reflect de novo fatty acid synthesis, elongation, and desaturation, as determined by mass isotopomer distribution analysis (MIDA) (Hellerstein and Neese 1992; Lee et al. 1998). Natural ^13^C abundance of the target and derivatizing compounds are routinely extracted for MIDA calculations (Lee 1996). For analysis of fatty acid synthesis, palmitate, and oleate from media or cell pellets were saponified with 30 % KOH and 100 % ethanol and extracted using petroleum ether. Fatty acids were converted to their methylated derivative using 0.5 N HCl in methanol and methyl palmitate and methyl oleate, dried, reconstituted in hexane and injected into the GC–MS and monitored at *m*/*z* 270, and *m*/*z* 264, respectively. Analyses of complete oxidation of [U-^13^C_6_]-fructose to ^13^CO_2_, were performed using 100 µl media samples in GC vials. To each sample was added 50 µl of 0.1 M NaHCO_3_ followed by 50 µl of 1.0 M HCl. The vials were immediately capped and placed on the GC/MS sample tray for analysis of the *m*/*z* 44 and *m*/*z* 45 ion group (Mizumori et al. 2006). Only ^13^C labeled product fractions, after their parent ion concentrations are resolved via standard Chemstation Integrator diagnostics following background subtraction, are reported. The internal standard is the ^13^C labeled fructose and its products as it gets metabolized following its addition at the time points of days 6 and 14 in adipocytes for a period of 48 h as described above. Glutamate was extracted from 100 µl media using 100 µl ultrapure water and 100 µl HPLC grade acetone. The mixture was frozen at −80 °C for 1 h, vortexed for 60 s and centrifuged at 13,000×*g* for 30 min. The supernatant was transferred into a 13 in. glass test tube on ice and dried under nitrogen. Glutamate was converted to its *N*-trifluoroacetyl-*n*-butyl (TAB) derivative in two steps (Leimer et al. 1977). Samples were mixed with 200 µl 3.0 M HCl in *n*-butanol and incubated at 100 °C for 1 h to form butyl esters. Samples were treated next with 100 µl dichloromethane and 25 µl trifluoroacetic anhydride at room temperature for 20 min to form TAB-glutamate and the products were dried under nitrogen. The residues were transferred into GC vials in 200 µl dichloromethane. When [U-^13^C_6_]-fructose is processed by glycolysis followed by pyruvate dehydrogenase (PDH), the [1,2-^13^C_2_]-acetyl-CoA produced is used to form [4,5-^13^C_2_]-glutamate. Alternatively, [3-^13^C_1_]-glutamate is produced from [U-^13^C_6_]-fructose when it is processed via the pyruvate carboxylase (PC) pathway which leads to [3-^13^C_1_]-oxaloacetate. Under EI conditions, ionization of TAB-glutamate produces two groups of fragments, *m*/*z* 198 *→* 202 and *m*/*z* 152 → 155, corresponding to the C2–C5 and C2–C4 fragments of glutamate (Bartnik et al. 2007). Therefore, detection of the ^13^C label on C4 and C5 of the *m*/*z* 200 M_+2_ glutamate fragment ion reflects PDH activity and hence oxidative phosphorylation (catabolic use of fructose). PC yields new (net) oxaloacetate for the cycle from fructose, which forms the anabolic (anaplerotic) substrate for the cycle. [1,2-^13^C_2_]-acetyl-CoA enrichment is calculated based on palmitate M_+4_/M_+2_ ratios as described previously (Lee et al. 1998). Flux surrogates are used throughout data interpretations, instead of flux calculations, whereby not (only) metabolite concentrations but their turnover rates are depicted by specific (positional) ^13^C labeling and their associations with fructose dosing. Figure 1 represents the potential ^13^C (red squares) labeled intermediates of lactate and glutamate that are formed from U^13^C_6_-D-glucose and described further in the “Results” section.

### Quantitative metabolite analysis

Media and cell pellet metabolic products were quantified and compared by integrated peak area values under corresponding total ion current (TIC) ID logs as determined by retention times in the selected ion monitoring mode by blinded spectra processing personnel (see Acknowledgments section). Average peak width values were less than 5 s (ChemStation Integrator, Agilent, Palo Alto, CA, USA) using identical injection volume, solvent strength and split ratios with that of natural ^13^C-labeled external chromatographic standards. Concentration-dependent integrated chromatographic TIC areas are displayed as arbitrary values of metabolite concentration among the control and fructose treated groups.

### Statistical analysis

Data are presented as arithmetic mean (average) plus standard error (SEM) of three independent observations using three independent integration results with background subtraction of the natural labeled ^13^C standard and its derivatizing agent. ANOVA with Dunnett’s test was used and *p* ≤ 0.05 was considered to indicate statistically significant differences in metabolite flux results in response to fructose treatments of adipocytes. Since this study examined fructose metabolism using a fructose tracer, the control used in this study comprised of cells treated with a very low concentration of 0.1 mM fructose. A linear regression analysis with *R* ^2^ (coefficient of determination) measurement was performed to describe the relationship between the fructose dosing and the SGBS adipocyte isotopomers measured. Correlation analysis was used to determine the strength of this association, and a *p* value ≤ 0.05 was used to indicate that the correlation is of statistical significance and different from 0.

## Results

### Fully differentiated adipocytes showed only a marginal increase in the oxidation of fructose

**Fig. 2:**
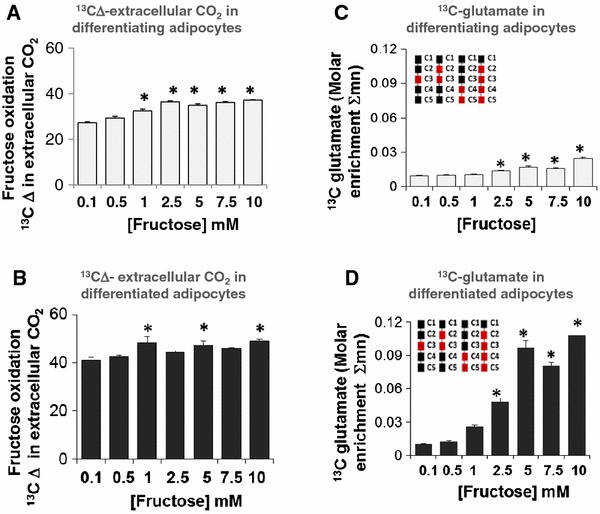
Fructose oxidation and fructose-derived glutamate synthesis via TCA cycle. Adipocytes were exposed to 0.1, 0.5, 1, 2.5, 5, 7.5 or 10 mM fructose in a medium containing a baseline amount of 5 mM glucose from the initiation of differentiation for 8 days (differentiating adipocytes) or 16 days (differentiated adipocytes). 10 % of fructose was supplied as [U-^13^C_6_]-fructose for a period of 48 h before harvest. Fructose oxidation was measured in differentiating adipocytes (**a**) and differentiated adipocytes (**b**) by the change Δ in the ^13^CO_2_ released in the extracellular medium measured as a ratio of ^13^C/^12^C (replacement of ^12^C by ^13^C) in extracellular bi-carbonate. ^13^C Δ is a measure of the ratio of stable isotopes ^13^C:^12^C, reported in parts per thousand (per mil,  ‰). Extracellular release of [^13^C]-glutamate in differentiating adipocytes (**c**) and differentiated adipocytes (**d**) is expressed as a percentage of total glutamate released

In an in vitro system, extracellular release of [^13^C]-HCO_3_ can be used to evaluate complete oxidation of [U-^13^C_6_]-fructose to CO_2_ via the TCA cycle following release of ^13^CO_2_ from [^13^C]-HCO_3_ using acid and alkali treatments. A measure of the ^13^CO_2_/^12^CO_2_ ratios (replacement of ^12^C by ^13^C) represents the change in ^13^C labeled extracellular CO_2_ released into the medium following treatment of cells with ^13^C labeled monosaccharide and reflects the complete oxidation of the monosaccharide through the TCA cycle. A slight but significant increase in released CO_2_, was observed in both differentiating (Fig. 2a) and differentiated adipocytes (Fig. 2b) at higher concentrations of fructose compared to the 0.1 mM fructose treatment. These results demonstrated that adipocytes take up fructose and metabolize it in the TCA cycle. However, this response is only marginally higher than the control and a robust dose-dependent response was not observed in adipocytes with increasing concentrations of fructose especially in differentiated adipocytes. This suggests that fructose does not serve as a robust substrate for energy production by complete oxidation and ATP synthesis. Compared to the differentiating adipocytes (Fig. 2a), the differentiated adipocytes (Fig. 2b) showed an overall higher level of extracellular CO_2_ release reflecting more complete oxidation of fructose in the differentiated adipocytes compared to differentiating adipocytes.

### Fructose increased extracellular glutamate indicating an increased anabolic function

Glutamate derived from the TCA cycle intermediate α-ketoglutarate provides a strategic byproduct for amino acid biosynthesis. When adipocytes were incubated with fructose, as described in the “Materials and methods” section, a significant increase in extracellular [^13^C]-glutamate was detected in cultures of both differentiating and fully differentiated adipocytes (Fig. 2c, d). The response was significant at fructose concentrations of ≥2.5 mM in differentiating (Fig. 2c) and in differentiated adipocytes (Fig. 2d). While the differentiating adipocytes only demonstrate a minor but significant increase in extracellular glutamate production, the response is very robust in differentiated adipocytes, with the 5 mM fructose treatment dose saturating and exhausting this flux with a sevenfold increase in [^13^C]-glutamate. Further increases in fructose concentration did not additionally augment the conversion of fructose to glutamate. These results also demonstrated the distinction between differentiating and differentiated adipocytes with respect to glutamate production. The increase in [^13^C]-glutamate produced from labeled fructose in differentiated adipocytes suggests a more prominent anabolic function in mature adipocytes.

### Fructose increased the PDH flux but decreased PC flux in adipocytes

Carbohydrates, including fructose, undergo glycolysis to form pyruvate as a metabolic intermediate. Pyruvate enters into the TCA cycle for anabolic processes or for energy production (catabolic process). It does so in one of two ways—either by conversion to acetyl-CoA via the PDH reaction, the catabolic pathway or by conversion to oxaloacetate via the PC reaction, to support gluconeogenesis, fatty acid synthesis and other anabolic processes. Using [U-^13^C_6_]-fructose enabled analysis of PDH or PC flux by measuring the glutamate isotopomers that are specific for each of these pathways.

**Fig. 3:**
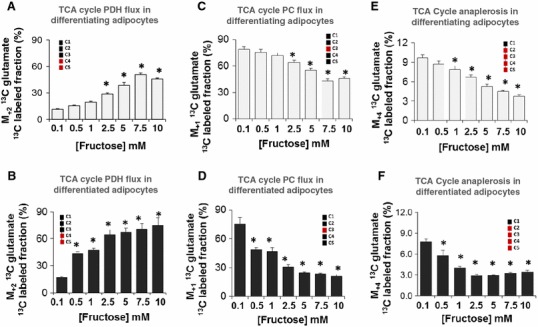
Entry of pyruvate into TCA cycle via PC or PDH flux and TCA cycle anaplerosis. Adipocytes were exposed to 0.1, 0.5, 1, 2.5, 5, 7.5 or 10 mM fructose in a medium containing a baseline amount of 5 mM glucose from the initiation of differentiation for 8 days (differentiating adipocytes) or 16 days (differentiated adipocytes). 10 % of fructose was supplied as [U-^13^C_6_]-fructose for a period of 48 h before harvest. PC flux was measured in differentiating (**a**) and differentiated (**b**) adipocytes as extracellular [3-^13^C_1_] glutamate using the M_+1_ ΣmC2–C5 fragment (one ^13^C substitution, *m*/*z* 199 fragment, EI) of its ^13^C labeled fraction. PDH flux was measured in differentiating (**c**) and differentiated (**d**) adipocytes as extracellular [4,5-^13^C_2_] glutamate using M_+2_ ΣmC2–C5 fragment (two substitutions, *m*/*z* 200 fragment, EI) of its ^13^C labeled fraction. TCA cycle anaplerosis was examined in differentiating (**e**) and differentiated (**f**) by measuring the M_+4_ glutamate flux (M_+4_ ΣmC2–C5) (four ^13^C substitution, *m*/*z* 202 fragment, EI). *p < 0.05 compared to the 0.1 mM fructose labeled. Please note that the first (C1) carbon of glutamate is excluded from EI analyses as this carbon may go through extensive exchange reactions

The C2–C5 *m*/*z* 200 M_+2_ glutamate fragment ion was used to measure PDH flux. The amount of [4,5-^13^C_2_]-glutamate formed from [U-^13^C_6_]-fructose is indicative of the PDH flux. C2–C5 M_+2_ labeled glutamate showed a robust dose-dependent increase in response to fructose in both differentiating (Fig. 3a) and differentiated adipocytes (Fig. 3b). The effect was more pronounced in the differentiated adipocytes in terms of magnitude of response and increased sensitivity in terms of dose. An increase in the PDH flux suggests that citrate was the primary product resulting from the entry of fructose into the TCA cycle.

[3-^13^C_1_]-glutamate is formed from [U-^13^C_6_]-fructose when the fructose-derived pyruvate intermediate is processed via the PC pathway and this was measured as C2–C5 *m*/*z* 199 M_+1_ glutamate fragment ion. A dose-dependent decrease in PC flux was observed in both differentiating (Fig. 3c) and differentiated adipocytes (Fig. 3d) in response to increasing fructose concentrations. Differentiated cells had a greater responsiveness compared to differentiating cells, similar to the PDH flux results. For example, exposure to 5 mM fructose showed a 1.4-fold decrease in the differentiating adipocytes while this decrease is 3-fold in the differentiated adipocytes. The decreases in PC flux further corroborates that fructose-derived pyruvate predominantly yields citrate via PDH.

### Fructose is not a source of TCA cycle anaplerosis in differentiating and differentiated adipocytes

Anaplerotic reactions, which are anabolic reactions that replenish the TCA cycle intermediates, occur constantly for efficient energy production and new citrate synthesis in the TCA cycle. Adipocytes exposed to increasing concentrations of fructose demonstrated a dose dependent decrease in the ^13^C M_+4_ glutamate. ^13^C M_+4_ glutamate is formed when PDH and PC reaction products combine to form newly labeled citrate. A key point is that PC derived oxaloacetate accepts tracer labeled acetyl-CoA obtained from fructose derived pyruvate via PDH which then forms glutamate in the TCA cycle. The ^13^C M_+4_ glutamate thus measures the anabolic use of fructose-derived pyruvate for oxaloacetate production and its condensation with fructose derived acetyl-CoA for the production of citrate. In differentiated adipocytes (Fig. 3f), 2.5 mM fructose maximally decreased TCA cycle anaplerosis with no further decrease in the formation of ^13^C M_+4_ glutamate at higher concentrations of fructose. The ^13^C M_+4_ glutamate was small and accounted for less than 10 % of the total ^13^C glutamate that is formed from fructose. Differentiating adipocytes (Fig. 3e) appeared to be less sensitive to fructose compared to the fully differentiated adipocytes (Fig. 3f). A decrease in ^13^C M_+4_ glutamate level supports previous observations that fructose was not an efficient substrate for maintaining energy production in the cycle. Previous ^13^C tracer studies with glucose (Liu et al. 2010) suggest that the glucose-derived pyruvate pool maintains TCA cycle anaplerosis in the presence of fructose.

### Fructose robustly increased intracellular palmitate, de novo palmitate synthesis and the release of palmitate from adipocytes

**Fig. 4:**
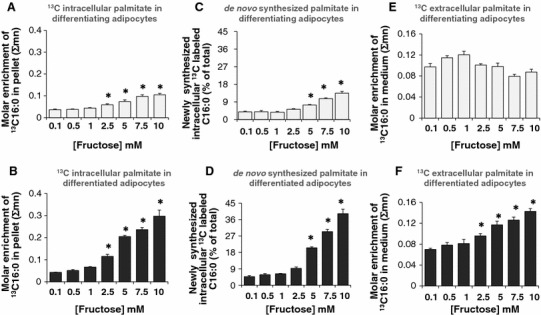
Fructose-induced synthesis and release of palmitate (C16:0) in adipocytes. Adipocytes were exposed to 0.1, 0.5, 1, 2.5, 5, 7.5 or 10 mM fructose in a medium containing a baseline amount of 5 mM glucose from the initiation of differentiation for 8 days (differentiating adipocytes) or 16 days (differentiated adipocytes). 10 % of fructose was supplied as [U-^13^C_6_]-fructose for a period of 48 h before harvest. ^13^C content (Σmn) of intracellular palmitate in differentiating (**a**) and differentiated (**b**) adipocytes. ^13^C labeled newly synthesized intracellular palmitate in differentiating (**c**) and differentiated (**d**) adipocytes, ^13^C content (Σmn) of extracellular palmitate in the medium form differentiating (**e**) and differentiated (**f**) adipocytes

Fructose-derived pyruvate enters the TCA cycle via the PDH flux or PC flux. Entry through the PDH flux results in the formation of acetyl-CoA that combines with oxaloacetate to form citrate. When excess citrate is formed, it can be shuttled out of the mitochondria and metabolized to form acetyl-CoA. In combination with malonyl-CoA, acetyl-CoA acts as a substrate for anabolic reactions of the lipogenesis pathway leading to the synthesis of fatty acids such as palmitate (C16:0). We found a statistically significant increase in intracellular [^13^C]-palmitate formed in differentiating (Fig. 4a) and differentiated adipocytes (Fig. 4b) exposed to increased levels of fructose. The increase in labeled palmitate was more pronounced in the differentiated adipocytes (Fig. 4b). These results demonstrated that fructose was robustly converted to palmitate in adipocytes and was therefore a potent lipogenic substrate in this cell type. The result was consistent with increasing mobilization of fructose towards fatty acid synthesis. Newly synthesized palmitate contributed only about 5 % of the total fatty acids at 0.1 mM fructose in both the differentiating (Fig. 4c) and fully differentiated adipocytes (Fig. 4d). A dose dependent increase in the newly synthesized palmitate occurred in adipocytes cultured in the presence of 5 mM or higher fructose (Fig. 4c, d). This response was more robust in differentiated adipocytes (Fig. 4d) which exhibited a two to ninefold increase in newly synthesized palmitate between 2.5 and 10 mM, supporting the conclusion that high concentrations of fructose increased lipogenesis.

Increasing concentrations of fructose did not significantly alter the release of palmitate compared to control in differentiating adipocytes (Fig. 4e). On the contrary, simultaneous with the increase in newly synthesized and labeled intracellular palmitate, differentiated adipocytes showed a dose-dependent increase in the release of palmitate to the extracellular medium (Fig. 4f). A 1.5–2.0-fold increase in the release of labeled palmitate from fully differentiated adipocytes was observed in cells treated with 2.5–10 mM fructose as compared to the 0.1 mM fructose treated group (i.e., the control). Overall, fructose increased intracellular palmitate formation in a robust manner in fully differentiated adipocytes while simultaneously also increasing the release of palmitate from the cells.

### Fructose increased intracellular oleate without altering the release oleate to the extracellular medium

**Fig. 5:**
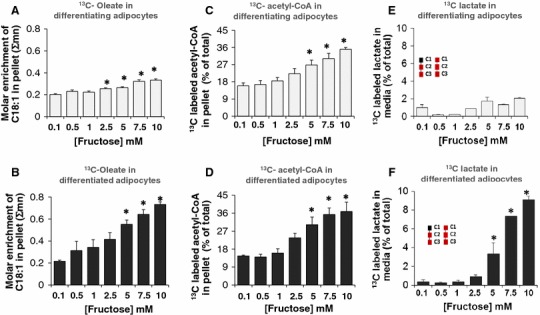
Fructose induced oleate (C18:1) synthesis, acetyl-CoA formation and lactate release from adipocytes. Adipocytes were exposed to 0.1, 0.5, 1, 2.5, 5, 7.5 or 10 mM fructose in a medium containing a baseline amount of 5 mM glucose from the initiation of differentiation for 8 days (differentiating adipocytes) or 16 days (differentiated adipocytes). 10 % of fructose was supplied as [U-^13^C_6_]-fructose for a period of 48 h before harvest. Intracellular oleate content in differentiating (**a**) and differentiated (**b**) adipocytes; ^13^C labeled intracellular acetyl-CoA in differentiating (**c**) and differentiated (**d**) adipocytes. ^13^C labeled lactate fraction as percent of total unlabeled lactate in the media in differentiating (**e**) adipocytes and in differentiated adipocytes (**f**), *p < 0.05 compared to the 0.1 mM fructose labeled

In addition to the increased intracellular synthesis of palmitate and its release, fructose-treated adipocytes also demonstrated an increase in the accumulation of intracellular oleate in differentiating (Fig. 5a) and differentiated (Fig. 5b) adipocytes. Oleate is formed by the elongation and desaturation of palmitate. These results imply that in addition to the activity of fatty acid synthase, fructose also increases the action of fatty acid elongase and fatty acid desaturase to bring about the conversion of palmitate to oleate thus promoting the storage of fatty acids as both oleate and palmitate. Oleate synthesis was distinctly higher in the differentiated adipocytes (Fig. 5b) compared to the differentiating adipocytes (Fig. 5a). No change was observed in the release of oleate into the medium in response to fructose treatment (data not shown).

### Fructose augmented acetyl-CoA formation and lactate release in adipocytes

Acetyl-CoA is the precursor for the synthesis of fatty acids including palmitate, and oleate. Fructose augmented the formation of [1,2-^13^C_2_]-acetyl-CoA from labeled fructose in both differentiating (Fig. 5c) and differentiated (Fig. 5d) adipocytes. About 15 % of the total pool of acetyl-CoA was comprised of tracer-labeled [1,2-^13^C_2_]-acetyl-CoA in the 0.1 mM fructose-treated adipocytes group. There was a dose-dependent increase in the tracer-labeled acetyl-CoA formed to 35–40 % in both differentiating (Fig. 5c) and differentiated (Fig. 5d) adipocytes. In adipocytes the acetyl-CoA pool was formed from the tracer labeled substrate at the expense of fatty acid breakdown via β-oxidation. The results also showed that isotopic enrichment of [1,2-^13^C_2_]-acetyl-CoA from labeled fructose was augmented in both differentiating and differentiated adipocytes and are evenly distributed in both. This indicates that increasing concentrations of fructose yield comparable acetyl-CoA in both differentiating and differentiated adipocytes. However, the acetyl-CoA formed may be utilized more in the differentiated adipocytes towards fatty acid synthesis since fatty acid formation was greater in the differentiated adipocytes compared to differentiating adipocytes (Fig. 4c, d).

Pyruvate enters into the TCA cycle under aerobic conditions to generate ATP via substrate-level and oxidative phosphorylation. Under anaerobic conditions, however, pyruvate can be converted to lactate which also results in energy production but at a much lower level. Examining the [^13^C]-lactate in the extracellular medium revealed that fully differentiated adipocytes but not differentiating adipocytes, showed show a dose-dependent release of lactate into the extracellular medium (Fig. 4e, f). The release was robust only at the higher concentrations of fructose (5, 7.5 and 10 mM) in differentiated adipocytes, suggesting that adipocytes efficiently produced lactate only in the presence of excess fructose.

Other potential metabolic endpoints for labeled fructose include the conversion to glycogen or ribose. In this study, fructose did not label ribose or glycogen efficiently (data not shown). This is consistent with the irreversible di-phosphorylation of fructose-6-phosphate which prevents its isomerization to glucose-6-phosphate and consequent metabolism towards glycogen and ribose in the pentose phosphate pathway.

### Fructose dose correlated significantly with the SGBS adipocyte isobolome (SGBS adipocyte specific ^13^C labeled metabolome)

**Fig. 6:**
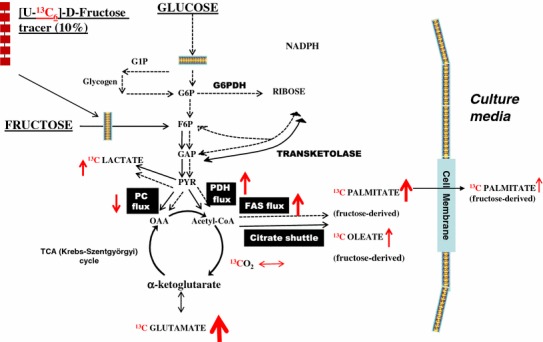
Schematic of the ^13^C labeled fructose-derived metabolites in adipocytes. Adipocytes were exposed to 0.1, 0.5, 1, 2.5, 5, 7.5 or 10 mM fructose in a medium containing a baseline amount of 5 mM glucose from the initiation of differentiation for 8 days (differentiating adipocytes) or 16 days (differentiated adipocytes). 10 % of fructose was supplied as [U-^13^C_6_]-fructose for a period of 48 h before harvest. The *red arrows* indicate the specific fold changes in response to 5 mM fructose treatment compared to the lowest treatment of 0.1 mM fructose (^13^CO_2_, +1.15×; extracellular [^13^C]-glutamate, +7.2×; PC flux, −0.32; PDH flux, +3.97×; intracellular [^13^C]-palmitate, +4.8×; extracellular release of [^13^C]-palmitate −1.67×; FAS flux, +4.33×; intracellular [^13^C]-oleate, +2.56×; extracellular [^13^C]-lactate, +3×). *G1P* glucose 1-phosphate, *G6P* glucose 6-phosphate, *G6PDH* glucose 6-phosphate dehydrogenase, *F6P* fructose 6-phosphate, *FAS* fatty acid synthase, *GAP* glyceraldehyde 3-phosphate, *PYR* pyruvate, *OAA* oxaloacetate. ↑ represents increasing fold change; ↓ decreasing fold changes and the thickness of the *arrow* represents intensity of the fold change

In order to determine how the [U-^13^C_6_]-fructose tracer-derived product isotopomers changed with respect to the different concentrations of fructose, multiple linear regression analyses were performed to determine the coefficients of determination (*R* ^2^) and correlation coefficients (*R*). Fructose exposure served as the independent variable and the ^13^C-labeled isotopomers were the dependent or response variables. Figure S1 represents the SGBS isobolome-wide associations in a heat map using the [U-^13^C_6_]-fructose flux surrogate markers with the corresponding fructose concentration. The flux surrogate markers are positional ^13^C labeled intermediary metabolic products that represent specific reactions and their rates in the isobolome (i.e., the ^13^C labeled metabolome). The values are the amount of the fructose-derived metabolites expressed as percentages of those levels detected in control adipocytes exposed to 0.1 mM labeled fructose on days 8 or 16, the differentiating (Fig. S1-A) or fully differentiated adipocytes (Fig. S1-B), respectively. The isotopomer association patterns for palmitate and oleate showed relatively high *R* ^2^ and *R* in differentiating (Fig. S1-A) and differentiated adipocytes (Fig. S1-B). These results indicated that the palmitate synthesis pathway and extracellular release of palmitate (limited to differentiated adipocytes) were strongly associated with the concentration of fructose in a significant manner and corroborate the lipogenic nature of fructose. For example, in differentiated adipocytes (Fig. 6b), the *R* ^2^ and *R* values were high and significant for [^13^C]-palmitate content (*R* ^2^ ≥ 0.950 and *R* ≥ 0.975), de novo palmitate synthesis via fatty acid synthase (*R* ^2^ ≥ 0.974 and *R* ≥ 0.987), intracellular [^13^C]-oleate content (*R* ^2^ ≥ 0.815 and *R* ≥ 0.903) and for the palmitate released (media [^13^C]-palmitate) ^13^C content) (*R* ^2^ ≥ 0.888 and *R* ≥ 0.943). However, *R* ^2^ and *R* values were low and not significantly correlated for the total cellular palmitate (peak area) in differentiated adipocytes (*R* ^2^ = 0.00 and *R* = 0.00) (Fig. S1-B) or inversely correlated in differentiating adipocytes (*R* ^2^ = 0.015 and *R* = −0.121) (Fig. S1-A). These results indicated that although palmitate was robustly labeled and generated from fructose, the fructose-derived intracellular palmitate is a transient product and is converted to oleate by elongation or released from the cells. Hence, the fructose-derived palmitate did not significantly alter the overall size of palmitate pool in adipocytes. On comparing the enrichment of palmitate and oleate (Figs. 5a, b, 6a, b), the enrichment of oleate is higher than that of palmitate. Oleate accumulation can be seen by the higher basal levels of labeled oleate compared to that of labeled palmitate in the control (0.1 mM fructose treated) adipocytes. Adipocytes store oleate-enriched triglycerides by elongating unlabeled palmitate into stearate, a process, as rapid as that of de novo palmitate synthesis. The increased oleate ^13^C labeling related to that of palmitate also indicates that palmitate is only partially, a direct precursor of newly labeled oleate. Since oleic acid gets readily incorporated into triglycerides as efficiently as its precursor (palmitate), palmitate’s elongation with fructose-derived acetyl-CoA into the stored product (oleic acid) is expected to occur in a lipogenic substrate environment, such as that observed in this study. However, based on regression analysis, palmitate enrichment correlated more strongly with increasing fructose concentrations compared to that with oleate, in this cell type (Fig. 1a, b) [(*R* ^2^ = 0.950 and *R* = 0.975) for palmitate enrichment in differentiated adipocytes and (*R* ^2^ = 0.907 and *R* = 0.952) in differentiating adipocytes] and [(*R* ^2^ = 0.815 and *R* = 903) for oleate enrichment in differentiated adipocytes and (*R* ^2^ = 0.850 and *R* = 0.922) in differentiating adipocytes respectively]. These results suggest that that there is also an attempt by the cell to simultaneously replenish the palmitate pool in the presence of increasing fructose concentrations as palmitate formed is being used up either for conversion to stearate and subsequently oleate or for release from adipocytes.

In addition to the fatty acid pathways, glutamate synthesis represented by extracellular [^13^C] media glutamate, ^13^C glutamate content (*R* ^2^ = 0.850 and *R* = 0.922) and isotopomers of the TCA cycle including PC flux (*R* ^2^ = 0.612 and *R* = −0.782) and PDH flux (*R* ^2^ = 0.590 and *R* = 0.768) showed strong and significant correlation with fructose dose. The correlation between fructose-derived lactate and the dose of fructose, representing the lactic acid fermentation pathway, showed statistical significance (*R* ^2^ ≥ 0.886 and *R* = 0.941) for extracellular [^13^C]-lactate content. Similarly, the correlation was significant for the association between fructose oxidation to carbon dioxide and the concentration of fructose (*R* ^2^ = 0.296 and *R* = 0.544) in differentiated adipocytes (day 16) with a stronger association with fructose (*R* ^2^ = 0.604 and *R* = 0.777) in differentiating adipocytes (day 8). Overall, these results support the conclusion that fructose was used as a substrate for oxidation in differentiating adipocytes but the primary use of fructose in differentiated adipocyte is for anabolic processes such as synthesis of fatty acids and non-essential amino acids.

Figure 6 is a schematic summarizing the features of fructose metabolism in adipocytes and the key metabolites derived from [U-^13^C_6_]-fructose in differentiated adipocytes treated with 5 mM fructose. Most of the labeled fructose entered the TCA cycle resulting in an increase in glutamate by 7.2-fold compared to the control. The majority of this fructose entry into the TCA cycle occurred via a nearly fourfold increased PDH flux, while the PC flux exhibits a decrease (−0.32-fold). The increased entry of fructose through the PDH flux resulted in (i) an increased shuttling of the fructose-derived acetyl-CoA and citrate into the fatty acid synthesis pathway, (ii) a 4.33-fold increase in fatty acid synthase and (iii) a resulting 4.8-fold increase in the fatty acid palmitate synthesized from fructose. A 2.56-fold increase was also seen in the formation of oleate. Although fatty acids, palmitate, and oleate were formed in response to fructose, only palmitate was released from the cells, a process augmented by 1.67-fold in presence of 5 mM fructose.

## Discussion

A number of potentially deleterious effects have been attributed to fructose consumption based on reports of the effects of fructose in rodent and human studies (Aeberli et al. 2011; Blakely et al. 1981; Elliott et al. 2002; Rizkalla et al. 1992; Stanhope 2012). These effects include de novo lipogenesis, hyperlipidemia, hyperuricemia, increased adiposity, a decrease in insulin sensitivity, and development of metabolic syndrome. Many of the effects of fructose can also result from its metabolism in extra-hepatic tissues.

The intermediary metabolism of fructose in the liver and hepatocytes is well described and is the subject of many articles and reviews (Mayes 1993; Tappy and Le 2010; Tappy et al. 2010). In addition to its effects on the liver, high fructose feeding in human subjects was also shown to augment VAT deposition and to increase the waist to hip ratio in response to fructose- but not glucose-sweetened beverages or foods (Aeberli et al. 2011). Similar increases in adiposity were also demonstrated in rodents fed with dietary fructose (Rizkalla et al. 1992) or fructose containing beverages (Jurgens et al. 2005). Long-term feeding studies (up to 6 months) with high fructose corn syrup showed abnormal weight gain and fat deposition in adipose tissue and an increase in circulating triglycerides in mice. The increased adiposity in animals was attributed to decreased lipolysis and decreased lipid mobilization (Rizkalla 2010). A few studies examined the metabolism of fructose in rat adipocytes and adipose tissue (Bellido and Herrera 1978; Froesch and Ginsberg 1962). However, the metabolism and the effects of fructose in human adipocytes had not yet been fully characterized.

The study reported here examined the metabolism of fructose in human adipocytes using the SIDMAP method that employed a uniformly labeled [U-^13^C_6_]-d-fructose as a tracer. The targeted tracer fate association method was used to analyze metabolic fluxes using flux surrogates. Human adipocytes were exposed to a broad range of fructose concentrations, based on most common dietary fructose exposure, to study the metabolic responses of cultured differentiating and differentiated adipocytes. The flow of substrate and products through the metabolic network was measured to better understand the metabolic fates of fructose in adipocytes.

The human SGBS preadipocyte cells (27) were used in this study owing to their similarities to isolated human adipocytes. SGBS cells are neither transformed nor immortalized. These cells can (i) proliferate for up to 50 generations with retained capacity for adipogenic differentiation (38), (ii) efficiently differentiate in the presence of PPARγ agonists, (iii) respond to insulin stimulation by increasing glucose uptake several fold, (iv) show catecholamine-stimulated lipolysis, and (v) exhibit lipolysis and apoptosis while expressing/secreting adipokines and express SLC2A5 the transporter specific for fructose transport (26). The reported fructose concentrations in systemic circulation range from 0.05 to 2 mM at basal levels (Hui et al. 2009; Munstedt et al. 2011; Wahjudi et al. 2010). Serum fructose concentrations as high as 17 and 10 mM fructose were reported to be measured 30 min and 2 h, respectively, after a fructose ingestion challenge in one study (Hui et al. 2009) and 7.2 and 3.6 mM when measured 1 and 2 h, respectively, in a second study (Munstedt et al. 2011). These results indicated that frequent consumption of sugar or fructose sweetened beverages and foods can thus maintain high levels of circulating fructose. Based on these human studies, differentiating and differentiated adipocytes were exposed to a wide range of fructose concentrations (0.1–10 mM), and the fructose-derived metabolic intermediates were examined. The experimental paradigm consisted of adding the different concentrations of fructose to a medium containing 5 mM glucose so as to closely mimic the normal physiological blood glucose concentrations which is about 5 mM. The range of fructose concentrations was chosen to cover most of the reported concentrations of fructose in systemic circulation and to understand the adipocyte metabolic response to fructose at these levels of exposure.

Carbohydrates are an important source for cellular energy and are preferentially used over lipids or proteins since carbohydrates are not efficiently stored in the body. The TCA cycle intermediates and products were analyzed since this is the hub for energy metabolism including the conversion of pyruvate to acetyl-CoA and subsequently to CO_2,_ producing energy in the form of ATP (Owen et al. 2002). From the metabolic intermediates evaluated in this study, fructose was metabolized via glycolysis and the TCA cycle to produce glutamate, fatty acids, CO_2_ and lactate. The responses to fructose were more pronounced in differentiated adipocytes compared to differentiating adipocytes. Although fructose-derived pyruvate was oxidized in the TCA cycle in differentiated adipocytes, these cells did not show a strong dose-dependent increase in CO_2_ production. However, at the same increasing concentrations of fructose, at which no significant changes in oxidation were observed, fatty acid synthesis occurred robustly and in a dose dependent manner. In addition, increasing concentrations of fructose up to 5 mM also robustly increased [4,5-^13^C_2_]-glutamate formation supporting another anabolic role for fructose. Glutamate is used in biosynthetic pathways leading to the formation of non-essential amino acids to supply increased protein for enzyme synthesis (Brosnan 2000). The changes in glutamate production were consistent with the adaptive structural changes that may occur with the formation of larger adipocytes with bigger lipid droplets to accommodate the excess fatty acids in response to fructose. These observations indicate that anabolic functions such as fatty acid formation and glutamate synthesis are prominent metabolic responses to fructose in differentiated adipocytes. Thus, fructose acts as an anabolic substrate for molecular synthesis and energy storage and much less for oxidation.

In this study, differentiating adipocytes underwent fructose oxidation along with weaker anabolic responses of increased total glutamate and palmitate synthesis. The results presented here indicate that the substrate and energy requirements in differentiating adipocytes exposed to fructose differ from those of fully differentiated adipocytes with cateplerotic process predominating to accommodate the metabolic changes occurring during the conversion of preadipocytes to mature adipocytes. Fructose had prominent effects on differentiating and differentiated adipocytes in this study. Fructose treatment induced adipogenesis in murine 3T3L1adipocytes (Du and Heaney 2012). While hypertrophic changes in adipocytes are demonstrated more conclusively, the occurrence of adipogenesis in adult adipose tissue is not fully established (Cristancho and Lazar 2011). In humans, adipose tissue is reported to undergo a 10 % annual turnover suggesting that adipogenesis occurs although at a small rate in adults, maintaining the adipose depot (Spalding et al. 2008). In vivo rodent studies have demonstrated an increase in cell numbers reflecting adipogenesis along with hypertrophic changes in cell size in response to over nutrition (Klyde and Hirsch 1979). Similar results have been observed in short term feeding studies in humans (Tchoukalova et al. 2010). The differences between differentiating and differentiated adipocytes in our studies suggest that the fructose overfeeding would lead to hypertrophic changes as a more prominent response. Larger adipocytes seen in obesity demonstrate higher and altered capacity in triglyceride accumulation and fatty acid efflux (Bjorntorp and Karlsson 1970).

When adipocytes were exposed to increasing concentrations of fructose, the PDH flux increased which resulted in increased pyruvate/citrate cycling in the TCA cycle. A primary product resulting from the increased PDH flux was citrate, which in excess was shuttled towards the fatty acid synthesis pathway. These results elucidate the lipogenic potential of fructose in adipocytes. Fructose-induced lipogenesis has been reported in liver and hepatocytes (Samuel 2011) and activation of PDH has been shown to be key regulatory step in this process in hepatocytes (Da Silva et al. 1992). Simultaneously, with the augmented PDH flux in adipocytes in this study, a dose dependent decrease was seen in the PC flux. A dose-dependent increase in released lactate was seen in the presence of high concentrations of fructose. These high fructose concentrations also were associated with increased FA synthesis. Others have shown that lactate was a significant precursor for triacylglycerol synthesis in adipocytes (Francendese and Digirolamo 1981). This study demonstrated that in adipocytes provided with excess fructose, palmitate was the primary fatty acid synthesized robustly by de novo fatty acid synthesis mediated by fatty acid synthase. In addition to palmitate, fructose also increased the formation of oleate resulting from the action of fatty acid elongase and desaturase that convert palmitate to oleate. An unsaturated fatty acid such as oleate is a preferred storage form of fatty acid in cells (Gavino and Gavino 1992; Kokatnur et al. 1992). Hence, fructose promotes the storage of fatty acids in adipocytes similar to that seen in hepatocytes.

In addition to augmenting the storage of fatty acids in adipocytes, fructose also enhances the release of FFA from adipocytes. Adipocytes release FFA by hydrolytic cleavage of triacylglycerols by the adipocyte specific hormone sensitive lipase or by the triacylglycerol hydrolase, Adipose triglyceride lipase, generating non-esterified fatty acids (Lass et al. 2011; Wang et al. 2008). In this study, the fructose-treated differentiated adipocytes dose-dependently increased the release of palmitate. However, fructose did not stimulate the release of oleate in adipocytes while it increased its formation suggesting that oleate is preferentially stored in adipocytes.

Adipocytes are localized in separate depots of adipose tissue most prominently in the VAT found in the intra-abdominal region and the subcutaneous adipose tissue (SAT) found peripherally (Lee, et al. 2013). Adipocytes are exposed to fructose via peripheral systemic circulation in the case of SAT or via the celiac or mesenteric arteries that supply the splanchnic/mesenteric bed associated with VAT. These arteries supply the intra-abdominal tissues, including intestinal tissue where fructose is absorbed, and anastomose extensively prior to emptying into portal vein (Andrew et al. 2005; Jacobson 1982; Parks and Jacobson 1985). The result of these anatomical and physiological processes is that VAT may be exposed to high concentrations of metabolites including fructose absorbed by the intestines prior to entering the portal vein that supplies the liver. Although the adipocytes used in this study are derived originally from SAT a similar response to fructose exposure by VAT may affect the liver due to the direct access via the portal vein, and hence would have systemic implications. While both SAT and VAT are associated with the development of metabolic syndrome, VAT is more strongly associated with the increased risk of developing hepatic insulin resistance and metabolic syndrome, while SAT can contribute to peripheral insulin resistance (Jensen 2008; Klein 2004). Increased FFA release from SAT can accumulate as intramyocellular lipids in ectopic tissues such as skeletal muscle leading to peripheral insulin resistance (Koutsari and Jensen 2006). Additionally, fructose-induced release of FFAs can cause lipotoxicity in the liver and pancreas. Non-alcoholic fatty liver disease, which can result from lipotoxicity of the liver, has also been shown to be associated with cardiovascular conditions such as cardiac lipotoxicity, ischemia, and diastolic dysfunction of the heart (Cusi 2012).

Taken together, the results presented here revealed that fructose can significantly augment the accumulation of fatty acids palmitate and oleate in differentiated adipocytes and simultaneously increase the release of palmitate from adipocytes. Fructose was not an efficient substrate for oxidation but was an anabolic substrate for glutamate and fatty acid synthesis. Conversion to lactate occurred only at very high fructose concentrations in this model. Thus, the formation and accumulation of fatty acids is one of the key consequences of fructose metabolism in adipocytes. Fructose-induced increase in adiposity can further trigger adipocyte dysfunction leading to altered secretion of fatty acids and adipokines and an increase in ER stress and inflammation (de Ferranti and Mozaffarian 2008) which can further accelerate the development of insulin resistance in addition to the lipotoxic effects of fructose.

## Electronic supplementary material

Below is the link to the electronic supplementary material.

Supplementary material 1 (DOC 57 kb)

## Abbreviations

SGBS: Simpson-Golabi-Behmel syndrome
TCA cycle: Tricarboxylic acid cycle
PC: Pyruvate carboxylase
PDH: Pyruvate dehydrogenase
FAS: Fatty acid synthesis
SIDMAP: Stable isotope based dynamic profiling
FFA: Free fatty acids
