# Supplementary material for: Metabolic fate of fructose in human adipocytes: a targeted 13C tracer fate association study
Source: Metabolomics. 2014 Aug 3;11(3):529–44. doi: 10.1007/s11306-014-0716-0 (PMC4419153; doi:10.1007/s11306-014-0716-0)
Supplement: Supplementary file 1 — Supplementary material 1 (DOC 57 kb) [file 11306_2014_716_MOESM1_ESM.doc]

**Supplementary Material**

Figure 1S: **Correlation of the SGBS adipocyte isobolome and the fructose treatment dose range.** SGBS adipocyte isobolome (representing the adipocyte ^13^C labeled metabolome) wide associations in a heat map using the [U-^13^C_6_]-fructose flux surrogates are examined for correlation with the dose of fructose in differentiating (day 8) (A) and differentiated (day 16) adipocytes (B). The figure represents the R^2^ (coefficient of determination, correlation coefficients (correl, R) and p-value for the correlation of the adipocyte ^13^C isotopomers and concentrations of fructose used for treatment. p<0.05 is considered to be significant. The darkest green in the heat map represents 100% with the lighter green shades representing decreasing % values and the brighter red shades representing increasing % values.
